# *Streptococcus pneumoniae* colonization in health care professionals at a tertiary university pediatric hospital

**DOI:** 10.1007/s10096-022-04446-z

**Published:** 2022-04-25

**Authors:** Lisa-Maria Steurer, Mathias Hetzmannseder, Birgit Willinger, Peter Starzengruber, Claudia Mikula-Pratschke, Andrea Kormann-Klement, Michael Weber, Angelika Berger, Agnes Grill

**Affiliations:** 1 Comprehensive Center for Pediatrics, Division of Neonatology, Pediatric Intensive Care and Neuropediatrics, Department of Pediatrics and Adolescent Medicine, Medical University of Vienna, Währingergürtel 18-20, Vienna, 1090 Austria; 2 Division of Clinical Microbiology, Department of Laboratory Medicine, Medical University of Vienna, Vienna, Austria; 3 Institute for Medical Microbiology and Hygiene, Austrian Agency for Health and Food Safety, Graz, Austria; 4 Department of Biomedical Imaging and Image-Guided Therapy, Medical University of Vienna, Vienna, Austria

**Keywords:** *Streptococcus pneumoniae*, Pneumococci, Pneumococcal carriage, Healthcare professionals, Pediatric hospital, Pneumococcal transmission, Pneumococcal serotypes

## Abstract

*Streptococcus pneumoniae* is a commensal of the human upper respiratory tract. In certain cases, it can lead to serious invasive infections peaking in very young children and the elderly. Especially young children are frequent carriers and are thus regarded as the reservoir for horizontal transmission of pneumococci. This is the first study evaluating pneumococcal colonization patterns in healthcare professionals working in a tertiary care pediatric hospital, including carriage prevalence, serotype distribution, and risk factors for carriage. One oropharyngeal and one nasal swab per individual were directly plated onto appropriate agar plates and conventional culture was used for bacterial identification. Pneumococcal isolates underwent serotyping using Neufeld’s Quellung reaction with type-specific antisera. Additional nasal and oropharyngeal swabs were taken for qPCR analysis targeting *lytA*. In total, 437 individuals were enrolled. *S. pneumoniae* was isolated in 4.8% (21/437) of the study cohort using conventional culture and in 20.1% (88/437) of subjects using qPCR. Independent risk factors for pneumococcal carriage were living in the same household with children under 8 years of age and being aged 36–45 years with a carriage prevalence reaching 11.6% (vs. 2.9%, *p* = 0.002) and 6.7% (vs. 4.3%, *p* = 0.029), respectively. The most common serotypes were 6C and 3. A total of 71.4% (15/21) of the detected serotypes are not included in any currently available pneumococcal vaccine; 28.6% (6/21) of the carried serotypes are included in the PCV13 vaccine. We found a relevant amount of pneumococcal carriage bearing the potential risk of horizontal in-hospital transmission.

## Introduction

Nasopharyngeal colonization with *Streptococcus pneumoniae* is of epidemiologic importance as it precedes disease and is the major source of the spread of the pathogen within the community [1]. The carriage prevalence is highest in young children, peaking in the first 2 years of life with carriage rates reaching 60% or more [2, 3]. After the age of 3–5, it gradually declines in most developed countries [4, 5].

Healthcare professionals (HCP) working in pediatrics are regularly exposed to respiratory secretions of young children who are regarded as the most common vector for horizontal pneumococcal transmission within a community [2, 4, 6] and at the same time to those patient cohorts with the highest risk for serious invasive pneumococcal disease. However, no data are available on pneumococcal carriage among asymptomatic health personnel in the pediatric sector.

The aim of this study, therefore, was to assess pneumococcal colonization patterns and serotypes in a large cohort of HCPs in a tertiary care pediatric hospital.

## Materials and methods

### Study design and participants

This study was conducted as a cross-sectional carriage survey evaluating *Streptococcus pneumoniae* colonization in asymptomatic HCPs at the Department of Pediatrics and Adolescent Medicine of the Medical University of Vienna during a 6-month study period from 20th April to 30th October 2018. It was nested on a meningococcal carriage study previously described [7]. All medical staff members working as nurses, doctors, or medical students were eligible for participation on voluntary basis. Exclusion criteria were antibiotic therapy in the preceding 4 weeks and working less than 4 weeks in pediatrics. Although not assessed formally, approximately 10–20% of eligible individuals refused participation.

### Specimen and data collection

Prior to sample collection, each volunteer signed a written informed consent form and was administered a de-identified self-administered questionnaire consisting of socio-demographic data and information on potential risk factors associated with pneumococcal carriage (all listed in Table 1).

**Table 1:** Participant characteristics: demographic characteristics of total sample (*n* = 437) and culture-detected *S. pneumoniae* carriers (*n* = 21)

**Category**		**Overall participants *n* (%)**	**Carriers *n* (%)**
*Total number*		437 (100%)	21/437 (4.8%)
*Profession*	Nurses	307 (70.3%)	12/307 (3.9%)
	Doctors	110 (25.2%)	7/110 (6.4%)
	Medical students	20 (4.5%)	2/20 (10%)
*Age group (years)*	18–25	91 (20.8%)	3/91 (3.3%)
	26–35	169 (38.7%)	12/169 (7.1%)
	36–45	89 (20.4%)	25/89 (6.7%)
	> 45	88 (20.1%)	0
*Main work setting*	Pediatric ward	175 (40.0%)	5/175 (2.9%)
	PICU	43 (9.8%)	0
	NICU/NIMCU	181 (41.4%)	13/181 (7.2%)
	Outpatient department	98 (22.4%)	8/98 (8.2%)
*Gender*	Female	374 (85.6%)	21/374 (5.6%)
	Male	63 (14.4%)	0
*Active smokers*		64 (14.6%)	3/64 (4.7%)
*Resp tract infections (*< *2 weeks)*		98 (22.4%)	7/98 (7.1%)
*Mean household size (persons)*		2.6 (1.2 SD), median 2.0 (1–8)	3.0 (1.1 SD), median 3.0 (1–6)
*Household with own children*	< 8 years	95/434 (21.9%)	11/95 (11.6%)
	8–13 years	55/434 (12.7%)	2/55 (3.6%)
	14–19 years	55/434 (12.7%)	1/55 (1.8%)
	No children	269/434 (62.0%)	8/269 (3%)
*Vaccination against S. pneumoniae (any serotype)*	Total sample	60/416 (14.4%)	2/60 (3.3%)
	Nurses	22/290 (7.6%)	1/22 (4.5%)
	Doctors	31/109 (28.4%)	1/31 (3.2%)
	Medical students	7/17 (41.2%)	0
*PCV13*	Total sample	27/413 (6.5%)	1/27 (3.7%)
*PPV23*	Total sample	17/413 (4.1%)	1/17 (5.9%)
*PCV13* + *PPV23*	Total Sample	5/413 (1.2%)	0

Standard sampling and detection methods were used according to the official recommendations of the WHO Pneumococcal Carriage Working Group [8]. As recommended for pneumococcal carriage studies in adults, the upper respiratory tract was sampled by taking swabs through the nose and from the oropharyngeal wall of each subject. Using a sterile flocked nylon swab (FLOQSwabs/ESwab™, Copan Diagnostics Inc., Brescia, Italy), two separate nasal and two separate oropharyngeal swabs per subject were collected by trained personnel. Instead of using strictly deep swabs from the posterior nasopharynx, nasal swabs as deep as easily achievable were taken instead and compared to the oropharyngeal swabbing site.

The first of each swab type was immediately plated onto Columbia Blood Agar (BD™ Columbia Agar with 5% Sheep Blood). The second nasal and oropharyngeal swabs were stored in Amies transport medium and transferred to the Austrian National Reference Center for Pneumococcal Disease in Graz (AGES) for pneumococcal detection by qPCR targeting conserved regions of the autolysin (*lytA*) gene [9]. Samples were considered positive for *S. pneumoniae* when the *lytA*-specific signal detected was ≤ 35 CT [10].

### Laboratory procedures and bacterial identification

The collected plates were processed at the Department for Clinical Microbiology of the Medical University of Vienna, where the media were incubated within 3 h at 37 °C in 5% CO2-enriched atmosphere and examined at 18–24 h and at 48 h for colonies of *S. pneumoniae*. Suspected colonies resembling pneumococcus were subjected to standard identification methods (i.e., gram staining, optochin sensitivity, and bile solubility testing).

All confirmed pneumococcal isolates subsequently underwent serotyping using Latex agglutination (ImmuLex™ Pneumococcus Kit, SSI Diagnostica) and the capsular swelling method (Quellung reaction) with type-specific antisera (SSI Diagnostica) at the Austrian National Reference Center for Pneumococcal Disease in Graz (AGES).

### Statistical analysis

All computations were performed using IBM SPSS Statistics for Windows version 23.0 (IBM, Armonk, NY).

Differences between the groups given categorical dependent variables were compared using two-sided Fisher’s exact test or chi^2^ test as appropriate [11]. In addition, Fisher’s exact tests were used to assess the correlation between risk factors and carriage rates. *p* values equal or below 5% were considered statistically significant. Subjects with missing information for a certain variable were excluded from analysis for that variable only.

## Results

### Participant characteristics and carriage prevalence

Full socio-demographic characteristics and pneumococcal vaccination history of all 437 participants are shown in Table 1.

Among the 437 oropharyngeal samples, 21 *S. pneumoniae* isolates were recovered by conventional culture, rendering an overall carriage rate of 4.8%. Nasal swabs were culture positive for *S. pneumoniae* in only 2 subjects whose oropharyngeal samples were also positive.

Using qPCR targeting *lytA*, pneumococci were detected in 20.1% (88/437) of participants by either oropharyngeal (17.6%; 77/437) and/or nasal swabbing site (3.7%; 16/437). However, 14.3% (3/21) of culture proven pneumococcal isolates did not have a positive PCR result targeting *lytA.*

An overview and comparison of all results using conventional culture and PCR in different swabbing sites is given in Table 2.

**Table 2:** Comparison of oropharyngeal vs. nasal sample site and conventional culture vs. PCR targeting lytA in detecting *S. pneumoniae* in healthy carriers

Laboratory method	Sample type			
	**Nasal**	**Oropharyngeal**	**Nasal + oropharyngeal**	**Positive any site**
**Culture pos**	2	21	2	21
**PCR pos**	16	77	5	88
**Culture + PCR pos**	2	17	2	18
**Positive any method**	16	81	5	91

### Risk factor analysis

All statistical analyses were performed on the basis of culture proven results and revealed that culture-detected carriage rates were significantly higher in subjects who stated to live in the same household with children under the age of 8 (11.6% vs. 2.9%; *p* = 0.002). Furthermore, the age groups 26–35 (7.1% carriers) and 36–45 (6.7% carriers) were more frequently colonized (*p* = 0.029). There was a significant association between the age group 26–35 and having children younger than 8 years of age. When corrected for this bias, solely living with children under 8 and the age group 36–45 were found to be independent risk factors for pneumococcal carriage in our study cohort.

We also noticed a trend towards females being more frequently carriers (21/374 vs. 0/63; *p* = 0.056), as all detected carriers were female and none of the participating men was found to be colonized with *S. pneumoniae*.

In contrast, there was no statistically significant association between carriage and all other parameters evaluated.

### Serotype distribution

**Fig. 1:**
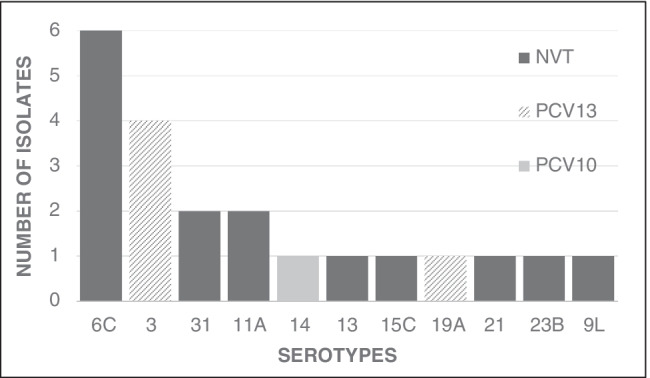
Serotype distribution: carried pneumococcal serotypes (*n* = 21) are summarized whether they are targeted by currently available 10-valent and 13-valent pneumococcal conjugate vaccines (**PCV10**: 1, 4, 5, 6B, 7F, 9 V, 14, 18C, 19F, and 23F; or **PCV13**: 1, 3, 4, 5, 6A, 6B, 7F, 9 V, 14, 18C, 19A, 19F, and 23F) or if they are non-vaccine types (NVT)

Twenty-three strains of 11 different serotypes were cultured from 21 of the 437 individuals analyzed in this study. In two subjects, isolates of the same serotype were cultured from both the oropharyngeal and nasal swabs and were hence considered to represent a single strain, reducing the overall number of strains cultured to 21. The most frequently carried serotype was 6C being detected in 28.6% (6/21) of colonized individuals, followed by the serotypes 3, 31, and 11A, which were found in 19.0% (4/21), 9.5% (2/21), and 9.5% (2/21) of carriers, respectively. An overview of the serotype distribution in the study cohort is shown in Fig. 1.

## Discussion

To our knowledge, this is the first study analyzing *S. pneumoniae* colonization patterns, serotype distribution, and contributing risk factors in a setting of asymptomatic healthcare personnel working in pediatrics and adolescent medicine.

The main finding of our study was an overall culture-based carriage prevalence of 4.8%, which was fourfold higher compared to 1.1% (38/3309) in a previous pneumococcal carriage study in Austria [12]. Compared to other recent pneumococcal carriage studies in Europe focusing on adults, we found a quite similar amount of carriage [13].

We might speculate to find higher carriage rates in individuals working in pediatrics than in the general population due to a higher exposure to pneumococcal disease and to respiratory droplets of colonized young children. However, direct comparison between studies is problematic and no comparable data on carriage in pediatric health care workers (HCW) are available up to date. Even evidence for HCW in general is currently limited to two published studies that directly compare pneumococcal carriage between HCW and non-HCW. Both studies report higher carriage rates in the HCW cohort [14, 15].

The strongest risk factor for carriage in our study cohort was to live in the same household with children under the age of 8 years. Carriage prevalence in this subcohort reached 11.6% (vs. 2.9%, *p* = 0.002). While more than half of our participants were childless non-elderly adults, 52.4% of all detected pneumococcal isolates were recovered from parents. This finding is not surprising against the background of children being regarded as the drivers of pneumococcal transmission and is well in line with previous studies [2, 4, 13, 16].

Our data show a relevant discordance between culture- and PCR-based results (4.8% vs. 20.1%). This finding is in concordance with recent studies reporting up to 15-fold higher pneumococcal detection in adults by qPCR compared to conventional culture particularly when oropharyngeal swabs were analyzed [13, 16, 17].

While WHO recommendations still include culture-based isolation of live pneumococci as gold standard for pneumococcal carriage studies, there is growing evidence that conventional non-enriched culture underestimates the true level of carriage as it has a good specificity but low sensitivity [13, 16–18]. The low sensitivity of carriage detection by culture in adults seems to result from the low density of pneumococcal colonization in asymptomatic carriers [19]. The human oropharynx is microbially rich which leads to bacterial overgrowth on culture plates making pneumococcal detection by culture difficult [20].

However, we might have overestimated *S. pneumoniae* carriage by qPCR targeting *lytA* only. Molecular detection of pneumococci targeting *lytA* is a recognized method with high sensitivity [9, 21], but it bears the risk of false positive results by a possible similarity between *lytA* and its homolog present in other streptococci colonizing the human nasopharynx [22–24]. It has therefore been recommended to use qPCR targeting more than one validated gene, such as *lytA* and *piaB* or *cpsA* [16, 17].

In addition, PCR may produce false positive results from interfering signals originating from nonviable bacteria.

Another aspect is that separate swabs were taken for culture and PCR analysis.

While sampling the nasopharynx is standard for pneumococcal carriage studies in children, there is ample evidence that oropharyngeal swabbing is superior in adults [13, 17, 25]. However, less is known on the efficacy of nasal swabs. Therefore, we intended to compare nasal swabs and oropharyngeal swabs/samples in their ability to detect asymptomatic pneumococcal carriage and found that nasal swabs were less sensitive than oropharyngeal samples regardless of the method used.

Regarding the serotype distribution, it is interesting that 85.7% of the carried serotypes were also found in patients with IPD in Austria during the study period [26]. We did not find carriage of vaccine serotypes in vaccinated individuals, albeit this study was not powered to detect interaction effects between pneumococcal carriage and immunization.

Of note, serotyping in our study was based on classical culture-based methods, which could have had a potential bias on serotype diversity. Recent studies reported that some serotypes/serogroups were only detected in qPCR positive samples [17].

This cross-sectional carriage survey has several limitations including the lack of a control group for direct comparison of results to non-healthcare individuals, as well as the lack of longitudinal data. The decision to forgo deep nasopharyngeal swabs and the amplification of *lytA* only instead of combining two validated genes for molecular pneumococcal detection are further drawbacks, as described above.

Despite several limitations, our study provides important new aspects and especially insight into the epidemiology of *S. pneumoniae* in a pediatric hospital setting. Our results show that asymptomatic pneumococcal colonization is common among HCP working in pediatrics bearing the potential risk of horizontal in-hospital transmission.

## Data Availability

The datasets generated during and/or analyzed during the current study are not publicly available to protect our participants’ sensitive data but are available from the corresponding author on reasonable request.
